# Identification and validation of core oxidative phosphorylation-related genes as biomarkers for immune response and diagnosis in burn injury

**DOI:** 10.3389/fimmu.2026.1818811

**Published:** 2026-08-12

**Authors:** Jiaqi Xu, Yujie Cui, Kangdi Li, Jia Jia, Xinfeng Huang

**Affiliations:** 1 Burn and Plastic Surgery, The Fifth Affiliated Hospital Sun Yat-sen University, Zhuhai, China; 2 Rheumatology, The Fifth Affiliated Hospital Sun Yat-sen University, Zhuhai, China

**Keywords:** ATP6V1C1, burn injury, ECHS1, immune microenvironment, LDHA, oxidative phosphorylation

## Abstract

**Introduction:**

Burn injuries are a common and devastating trauma that induce profound alterations in the immune microenvironment and further influence patient recovery and clinical outcomes. This study aimed to characterize the immune landscape of burn patients and identify key biomarkers for early diagnosis and targeted therapeutic strategies.

**Methods:**

Transcriptomic data from the GSE37069 and GSE19743 datasets were used for differential expression analysis to screen oxidative phosphorylation-related genes. Three machine learning algorithms (SVM-RFE, Random Forest, and Lasso) were combined to shortlist core genes. The differential expression of candidate genes was validated by qPCR and Western blot in burn tissues. Immune cell infiltration profiles were analyzed with the CIBERSORT algorithm. Correlation analysis was performed between core genes and immune cell subsets. Gene silencing assays in LPS-stimulated THP-1–derived macrophages were conducted to explore functional mechanisms, and a diagnostic nomogram was constructed to evaluate diagnostic performance.

**Results:**

A total of 23 oxidative phosphorylation-related differentially expressed genes were identified. Three core genes — ATP6V1C1, ECHS1, and LDHA — were screened out and confirmed to be significantly associated with burn progression. Immune infiltration analysis revealed distinct immune cell compositions between two patient groups: patients in group A exhibited a more active immune response with increased levels of B cells, M0 macrophages, and CD4+ memory T cells, while group B patients had higher proportions of CD8+ T cells and resting NK cells, indicating a more tolerogenic immune state. Correlation analysis suggested that ECHS1 may be involved in modulating immune cell activity, whereas ATP6V1C1 and LDHA may participate in the early immune response. In vitro validation showed that knockdown of ATP6V1C1 or LDHA markedly attenuated the secretion of pro-inflammatory cytokines (IL-6, TNF-α, IFN-γ), as well as mitochondrial reactive oxygen species production and oxygen consumption; by contrast, ECHS1 knockdown further amplified the inflammatory response. The diagnostic nomogram based on the three core genes demonstrated excellent diagnostic performance.

**Discussion:**

These findings provide valuable insights into the immunological dynamics of burn patients. The identified core genes highlight potential molecular targets for improving prognosis and therapeutic outcomes, and the nomogram offers a promising tool for burn patient stratification and personalized treatment.

## 1 Backgrounds

Burn injuries are one of the most common and devastating types of trauma worldwide, which are characterized by severe tissue damage, significant systemic inflammatory responses, and high rates of morbidity and mortality (1). Annually, millions of burn patients require medical treatment, with the mortality rate for severe burns exceeding 35% (2). These injuries not only have a profound impact on patients’ physiological functions and quality of life, but also impose a heavy burden on healthcare systems. Despite continuous improvements in comprehensive treatment measures such as fluid resuscitation, anti-infection therapy, and wound management, early diagnosis and accurate assessment of burn severity remain major challenges in clinical management. Due to the lack of sensitive and specific biological markers, the clinical judgment of burn severity is primarily based on experience and traditional scoring systems, which are insufficient for personalized treatment. Therefore, identifying molecular biomarkers that can early detect burn status and reflect pathological changes is of significant importance for guiding precision treatment and improving patient prognosis (3).

After a burn injury, the body undergoes complex pathophysiological changes with core features including extensive local tissue destruction, systemic inflammatory response syndrome (SIRS), and multiple organ dysfunction syndrome (MODS) (4). Local tissue damage leads to increased vascular permeability and edema, while the systemic inflammatory response triggers immune system dysregulation, enhanced oxidative stress, and metabolic abnormalities. Cytokine storms, leukocyte activation, and endothelial dysfunction collectively contribute to the ongoing tissue damage associated with burns (5). Furthermore, the immune response after burn injury is biphasic with early excessive inflammation followed by late-stage immune suppression, further increasing the risk of infection and organ failure. The molecular mechanisms underlying these pathological processes are complex and have not been fully elucidated (6).

Oxidative phosphorylation (OXPHOS) is the principal mitochondrial pathway of cellular energy production, and it has increasingly been recognized as a key determinant of immune cell activation, differentiation, and effector function (7). Following burn injury, the body mounts stress and inflammatory signaling cascades, including the NF-κB, MAPK, and JAK/STAT pathways, which drive the release of inflammatory mediators and the activation of immune cells (8–10). These processes are tightly coupled to mitochondrial energy metabolism: excessive inflammation and endothelial injury are accompanied by disturbances of mitochondrial homeostasis and oxidative phosphorylation activity, promoting oxidative stress, metabolic dysregulation, and the development of multiple organ injuries (11). A systematic analysis of the oxidative phosphorylation–related regulatory network in burns may therefore deepen our understanding of the underlying molecular pathology and provide new approaches for identifying early diagnostic biomarkers and potential therapeutic targets. It should be emphasized that oxidative phosphorylation, a mitochondrial metabolic pathway, is a concept distinct from protein phosphorylation, a post-translational modification; the present study focuses specifically on oxidative phosphorylation–related genes.

Based on this, the present study aims to comprehensively analyze transcriptomic data and oxidative phosphorylation–related genes (OXPHOS-related genes) to identify key OXPHOS-related genes closely associated with burn injury. The study will investigate their biological functions and potential signaling pathways, and further construct a burn diagnostic model based on OXPHOS-related gene features. The model will be validated and evaluated using machine learning algorithms, with the goal of providing new molecular evidence for the early diagnosis of burns and offering insights into precision treatment and disease risk stratification.

## 2 Materials and methods

### 2.1 Data collection

Gene expression datasets of burn patients, GSE37069 (comprising 553 burn samples and 37 control samples) and GSE19743 (comprising 114 burn samples and 63 control samples), were downloaded from the GEO database. The GSE37069 dataset was used as the training set for model development, while GSE19743 served as an independent external validation set. The oxidative phosphorylation gene set was obtained from the GSEA/MSigDB website (https://www.gsea-msigdb.org/gsea/msigdb/human/search.jsp), and the gene set name was HALLMARK_OXIDATIVE_PHOSPHORYLATION.v2025.1.Hs. It should be noted that both GSE37069 and GSE19743 consist of longitudinal whole-blood samples obtained at multiple time points from a limited number of severely burned patients rather than a single sample per subject (for example, GSE19743 comprises 114 arrays from 57 patients sampled at two time points, together with 63 healthy controls); this non-independence of samples was taken into account when interpreting the diagnostic performance and is addressed in the Discussion.

### 2.2 Human sample collection

This study was approved by the Ethics Committee of The Fifth Affiliated Hospital Sun Yat-sen University (2026 K72-1). All patients or their guardians provided informed consent for the collection of tissue samples in accordance with the principles outlined in the Declaration of Helsinki. The samples were collected under ethical guidelines to ensure patient privacy and confidentiality. The inclusion criteria for burn patients were based on clinical diagnoses of burn injuries, while healthy control samples were obtained from patients undergoing routine surgeries with no history of infection, malignancy, or immune disorders. The tissue samples collected included both burned tissue and adjacent normal tissue, which were immediately processed for further analysis. All patient data were anonymized to protect their identity and personal information. For experimental validation, burn tissues and paired adjacent normal skin were collected from 20 patients with second- to third-degree burns, and healthy control skin was obtained from 20 individuals undergoing elective plastic surgery without a history of infection, malignancy, or immune disorders. The detailed demographic and clinical characteristics of the enrolled subjects—including age, sex, burn depth, percentage of total body surface area (%TBSA) burned, time from injury to sampling, anatomical site, and infection status—are summarized in **Supplementary Table 1**.

### 2.3 Differential gene identification and gene screening

Differential expression analysis was performed on the GSE37069 dataset using the limma package in R, with thresholds set at |log2FC| > 1 and P-value < 0.05. A volcano plot was generated using the online tool (https://www.bioinformatics.com.cn, accessed on December 10, 2024), and Venn diagram analysis was employed to identify differentially expressed genes associated with oxidative phosphorylation for subsequent research.

### 2.4 Machine learning-based core gene screening

To further identify core oxidative phosphorylation genes, three machine learning algorithms including SVM-RFE, RF, and Lassowere applied. A Venn diagram was used to determine the intersection of genes selected by the three algorithms. Lasso regression analysis was performed using the glmnet package, with 10-fold cross-validation to determine the optimal penalty parameter (λ) and to select genes with non-zero coefficients. The caret and tidyverse packages were used to perform SVM-RFE analysis, with radial basis function (RBF) kernel and 10-fold cross-validation to evaluate feature subset performance. Random forest analysis was conducted using the randomForest package, constructing 500 decision trees and evaluating gene importance based on the average decrease in accuracy. Finally, pROC package was used to generate ROC curves for core genes and compute the area under the curve (AUC) to assess diagnostic performance.

### 2.5 The construction of nomogram

Based on the expression levels of the three core oxidative phosphorylation genes, a nomogram was constructed using the rms package. The ROC curve AUC was used to evaluate the role of each key gene and the nomogram model in the diagnosis of burn patients. Calibration curves and decision curve analysis were performed using the rmda package to evaluate the predictive efficiency of the nomogram for burn patients. In addition, the three core genes were entered into a multivariable logistic regression model, and the odds ratio (OR), 95% confidence interval (CI), and P value of each gene were calculated to determine whether each gene served as an independent diagnostic factor (**Supplementary Table 2**).

### 2.6 Consensus clustering analysis

Consensus clustering was performed using the ConsensusClusterPlus package with the K-means algorithm, based on the expression levels of the three core oxidative phosphorylation genes. To ensure stability and consistency, the optimal number of clusters was determined after 1000 iterations, resulting in the classification of burn patients into two groups.

### 2.7 Gene set enrichment analysis

Differential expression analysis between Burn group A and Control group B patients was performed using the limma package, and genes were ranked according to log2FoldChange. GSEA was subsequently performed using the ClusterProfiler package, based on the KEGG gene sets from the MSigDB database (https://www.gsea-msigdb.org/gsea/msigdb). The top six significantly upregulated and downregulated pathways were extracted for further analysis. Results were visualized using the ggstatsplot package.

### 2.8 Immune infiltration analysis

Immune cell infiltration levels in burn patients were assessed using the CIBERSORT package. The ggplot2 package was used to plot bar charts depicting the immune infiltration levels in each patient. Wilcoxon tests were performed to statistically compare the differences in the infiltration of 22 immune cell types between groups. Furthermore, Spearman’s rank correlation coefficients were calculated to explore the correlation between the expression levels of diagnostic biomarkers and immune cell infiltration.

### 2.9 quantitative PCR

For gene expression analysis, burn and normal tissue samples were collected from burn patients and healthy controls. Total RNA was extracted using TRIzol reagent (Invitrogen, USA) following the manufacturer’s protocol. RNA concentration and purity were assessed with a NanoDrop spectrophotometer, and cDNA was synthesized using the PrimeScript RT reagent Kit (Takara, Japan). qPCR was performed on a QuantStudio 5 Real-Time PCR System (Applied Biosystems, USA) using SYBR Green PCR Master Mix (Takara, Japan), with β-Actin as the reference gene. Specific primers for ATP6V1C1, ECHS1, and LDHA were designed using Primer-BLAST (NCBI). The relative gene expression was calculated using the 2^-ΔΔCT method.

### 2.10 Western blot analysis

For Western blot (WB) analysis, proteins were extracted using RIPA lysis buffer (Beyotime, China) containing protease inhibitors. Protein concentrations were quantified using the BCA Protein Assay Kit (Thermo Fisher Scientific, USA). Equal amounts of protein (30 µg) were separated by SDS-PAGE, transferred to PVDF membranes, and blocked with 5% non-fat milk in TBS-T buffer for 1 hour. Membranes were incubated overnight at 4 °C with primary antibodies against ATP6V1C1, ECHS1, LDHA, and β-Actin (Proteintech, China). After washing, membranes were incubated with HRP-conjugated secondary antibodies for 1 hour at room temperature. The proteins were detected using the ECL detection system (Thermo Fisher Scientific, USA), and band intensities were quantified with ImageJ software (NIH, USA). The band intensity of each target protein was normalized to that of β-Actin to determine the relative protein expression level.

### 2.11 Cell culture, macrophage differentiation and LPS stimulation

The human monocytic cell line THP-1 was cultured in RPMI-1640 medium supplemented with 10% fetal bovine serum and 1% penicillin–streptomycin at 37 °C in a humidified atmosphere containing 5% CO2. To induce differentiation into adherent macrophages, THP-1 cells were treated with 100 ng/mL phorbol 12-myristate 13-acetate (PMA) for 24 h. After a further 24 h of rest, the differentiated macrophages were stimulated with lipopolysaccharide (LPS, 100 ng/mL) for 24 h to establish an *in vitro* inflammatory model mimicking the burn-induced immune activation of macrophages.

### 2.12 Small interfering RNA transfection

For each of the three core genes (ATP6V1C1, ECHS1, and LDHA), three independent siRNAs and a non-targeting negative control (NC) were synthesized. PMA-differentiated THP-1 macrophages were transfected with siRNA using Lipofectamine RNAiMAX (Invitrogen, USA) according to the manufacturer’s instructions. Knockdown efficiency was verified by qPCR 48 h after transfection, and the siRNA with the highest silencing efficiency for each gene was selected for subsequent functional assays. The siRNA sequences are listed in **Supplementary Table 3**.

### 2.13 Enzyme-linked immunosorbent assay

Cell culture supernatants were collected after LPS stimulation, and the concentrations of secreted interleukin-6 (IL-6) and tumor necrosis factor-α (TNF-α) were measured using commercial ELISA kits (Human IL-6 ELISA Kit and Human TNF-α ELISA Kit) according to the manufacturer’s protocols. Absorbance was recorded at 450 nm with a microplate reader, and cytokine concentrations were calculated from standard curves.

### 2.14 Measurement of intracellular reactive oxygen species

Intracellular ROS levels were assessed using the fluorescent probe 2′,7′-dichlorodihydrofluorescein diacetate (DCFH-DA; Beyotime, China). After the indicated treatments, cells were incubated with DCFH-DA at 37 °C for 20 min in the dark and washed with serum-free medium, and the mean fluorescence intensity (MFI) was quantified by flow cytometry.

### 2.15 Measurement of mitochondrial oxygen consumption rate

Mitochondrial respiration was evaluated with the Seahorse XF Cell Mito Stress Test on a Seahorse XF extracellular flux analyzer (Agilent, USA). The OCR was recorded under basal conditions and after the sequential injection of oligomycin, carbonyl cyanide-4-(trifluoromethoxy)phenylhydrazone (FCCP), and rotenone/antimycin A. Basal respiration, maximal respiration, ATP production, and spare respiratory capacity were derived from the OCR profiles as described previously.

### 2.16 Statistical analysis

Statistical analyses were performed using GraphPad Prism 10 and R software. For the transcriptomic datasets, differential expression between groups was analyzed with the limma package (moderated t test), differences in immune cell infiltration were compared using the Wilcoxon rank-sum test, and Spearman’s rank correlation was used for correlation analyses. For the *in vitro* experiments, data are presented as the mean ± standard deviation (SD) of at least three independent replicates. Comparisons between two groups were performed using the two-tailed Student’s t test, and comparisons among multiple groups were performed using one-way analysis of variance (ANOVA) followed by Tukey’s *post hoc* test. A P value < 0.05 was considered statistically significant (ns, not significant; *P < 0.05; **P < 0.01; ***P < 0.001).

## 3 Results

### 3.1 Screening of oxidative phosphorylation-related genes in burn patient transcriptomes

**Figure 1:**
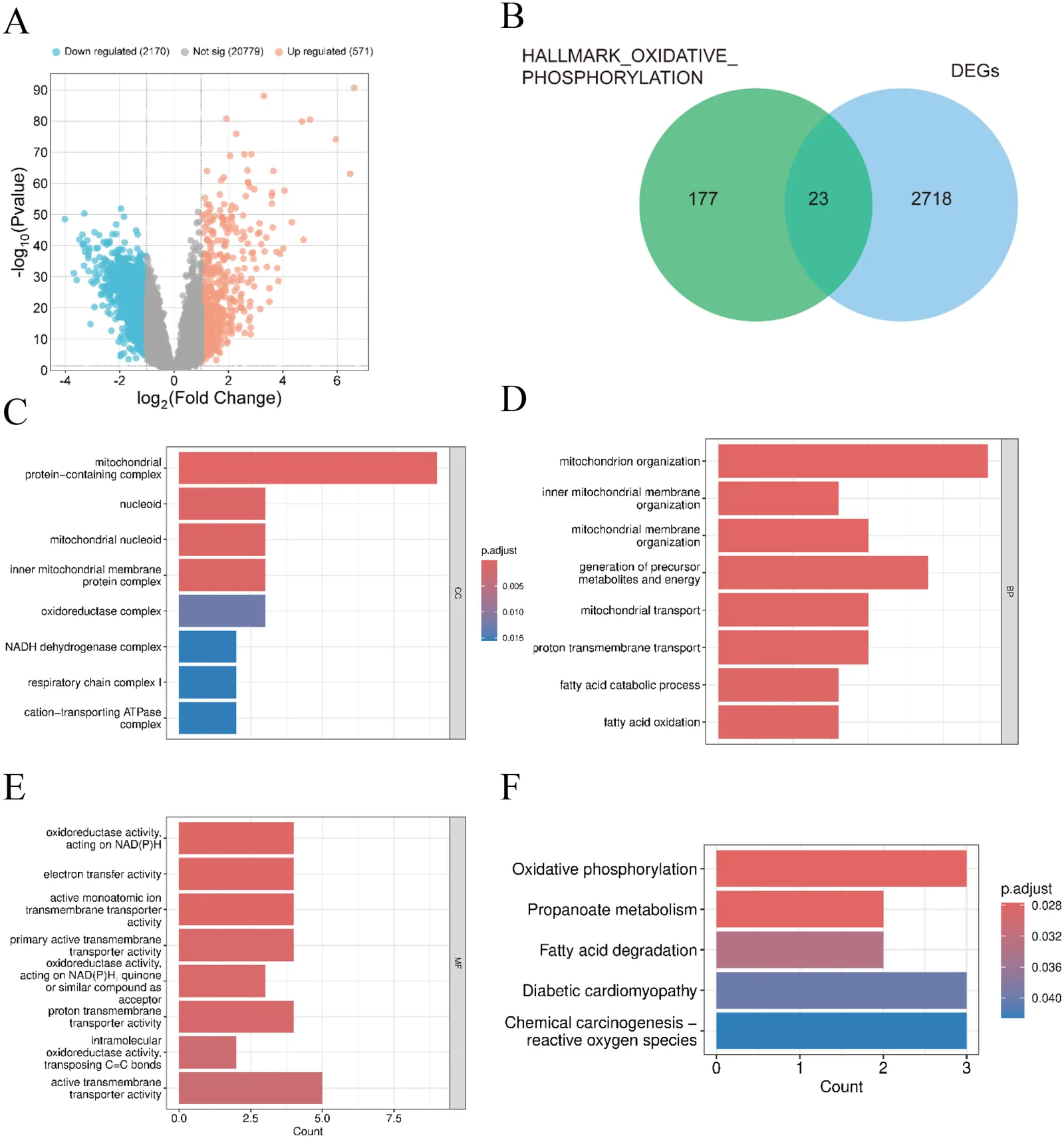
Identification and functional enrichment of oxidative phosphorylation-related genes in burn patients. **(A)** Volcano plot showing differential expression analysis of normal vs. burn patient samples from the GSE37069 dataset, highlighting genes with |log2FC| > 1 and p-value < 0.05. **(B)** Venn diagram showing the intersection between oxidative phosphorylation-related genes from MSigDB and differentially expressed genes, resulting in 23 candidate genes. **(C)** GO cellular component analysis of the 23 candidate genes, with key terms including “mitochondrial protein−containing complex” and “mitochondrial nucleoid”. **(D)** GO biological process analysis highlighting pathways like “mitochondrion organization” and “mitochondrial membrane organization”. **(E)** GO molecular function analysis showing enrichment in “oxidoreductase activity” and “electron transfer activity”. **(F)** KEGG pathway analysis revealing significant enrichment in “Oxidative phosphorylation”, “Propanoate metabolism”, and “Fatty acid degradation”.

First, differential expression analysis was performed on the GSE37069 dataset, comparing normal and burn patients. The thresholds for differential analysis were set as |log2FC| > 1 and P-value<0.05, resulting in the identification of 2,741 significant differentially expressed genes, including 2,170 downregulated genes and 571 upregulated genes (**Figure 1A**). Next, the oxidative phosphorylation gene set HALLMARK_OXIDATIVE_PHOSPHORYLATION.v2025.1.Hs, which includes 200 oxidative phosphorylation-related genes, was downloaded from the MSigDB website. Venn diagram analysis was then performed to intersect the oxidative phosphorylation genes with the differentially expressed genes, resulting in 23 candidate oxidative phosphorylation-related genes (**Figure 1B**). These candidate genes were further subjected to GO and KEGG functional enrichment analysis.

In the GO cellular component analysis, these candidate genes were predominantly associated with terms such as “mitochondrial protein−containing complex”, “nucleoid”, and “mitochondrial nucleoid” (**Figure 1C**), indicating their close association with mitochondrial function. Similarly, in the GO biological process analysis, enriched pathways included “mitochondrion organization”, “inner mitochondrial membrane organization”, and “mitochondrial membrane organization” (**Figure 1D**), further supporting the key role of these genes in regulating mitochondrial function. In the GO molecular function analysis, the main enriched pathways included “oxidoreductase activity, acting on NAD(P)H” and “electron transfer activity” (**Figure 1E**), further highlighting the central role of oxidative phosphorylation in cellular energy metabolism. KEGG enrichment analysis revealed significant differences in pathways such as “Oxidative phosphorylation”, “Propanoate metabolism”, and “Fatty acid degradation” (**Figure 1F**), further demonstrating the potential impact of oxidative phosphorylation in burn patients.

Selection of core oxidative phosphorylation genes using multiple machine learning algorithms.

**Figure 2:**
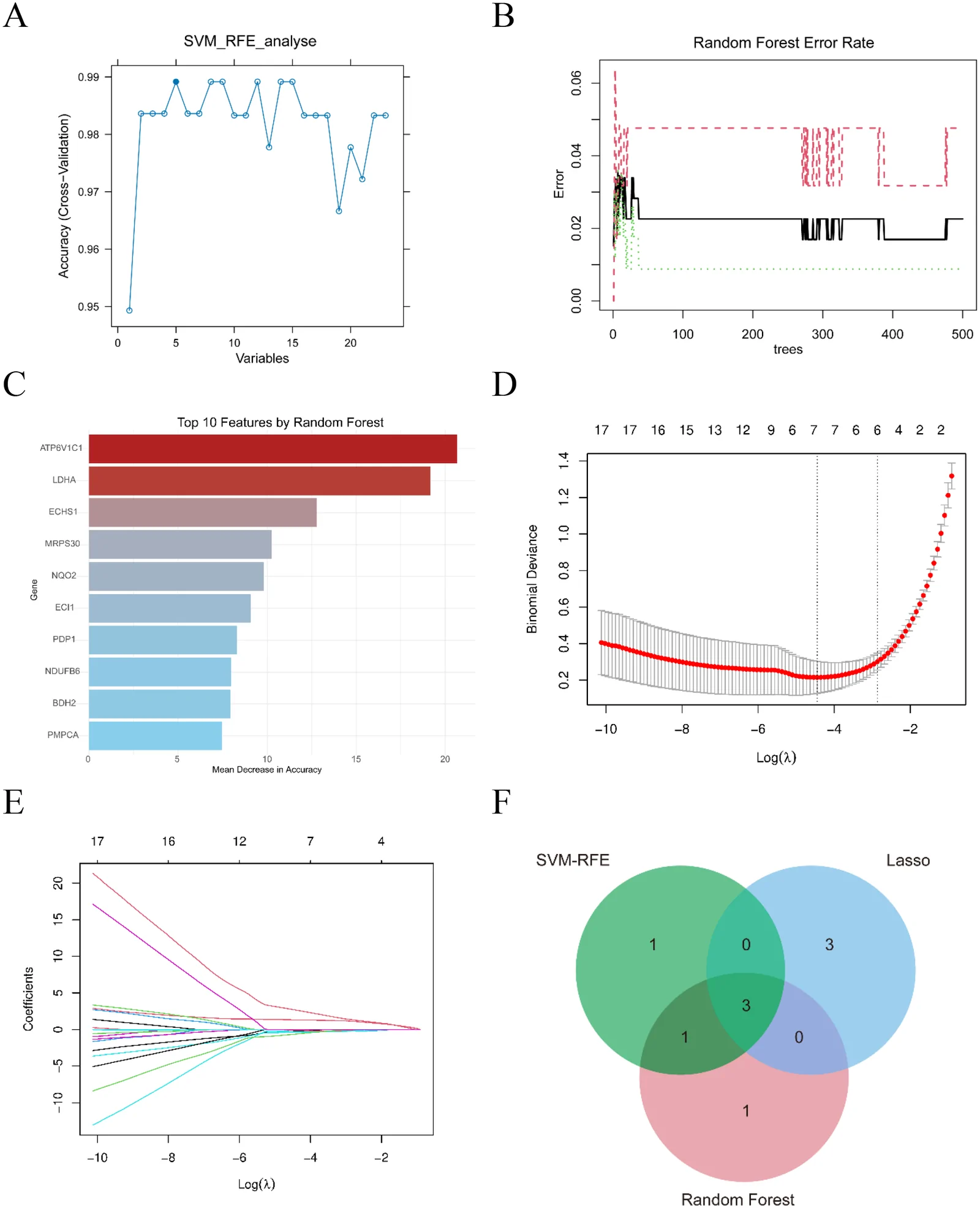
Core oxidative phosphorylation genes identified by multiple machine learning algorithms. **(A)** SVM-RFE algorithm identifying five key genes from the 23 candidate oxidative phosphorylation genes. **(B-C)** Random Forest (RF) algorithm ranking the importance of the 23 genes in burn diagnosis, with the top five genes showing high predictive value. **(D)** Lasso regression narrowing down the 23 candidate genes to 6 key genes. **(E)** Venn diagram showing the intersection of genes selected by SVM-RFE, RF, and Lasso, revealing three core genes: ATP6V1C1, ECHS1, and LDHA.

Given that the 23 candidate oxidative phosphorylation genes may play key roles in the progression of burn injuries, we further screened these genes using three machine learning algorithms: Lasso, SVM-RFE (Support Vector Machine Recursive Feature Elimination), and Random Forest (RF). Each of these algorithms has unique strengths in feature selection and high-dimensional data processing, complementing each other to more precisely identify core genes related to burns. First, the SVM-RFE algorithm identified five key genes from the 23 candidates, which showed strong predictive power for burn patient diagnosis (**Figure 2A**). Similarly, the Random Forest (RF) algorithm ranked the importance of the 23 candidate genes in burn diagnosis, with the top five genes demonstrating high predictive value (**Figure 2B, C**). Additionally, Lasso regression further reduced the 23 genes to 6 key genes (**Figures 2D–F**). To identify common core genes, we performed Venn diagram analysis of the genes selected by all three algorithms. Ultimately, three core genes were identified: ATP6V1C1, ECHS1, and LDHA (**Figure 2E**). These genes are considered critical in the diagnosis and progression of burn injuries.

### 3.2 Validation and functional analysis of core genes

**Figure 3:**
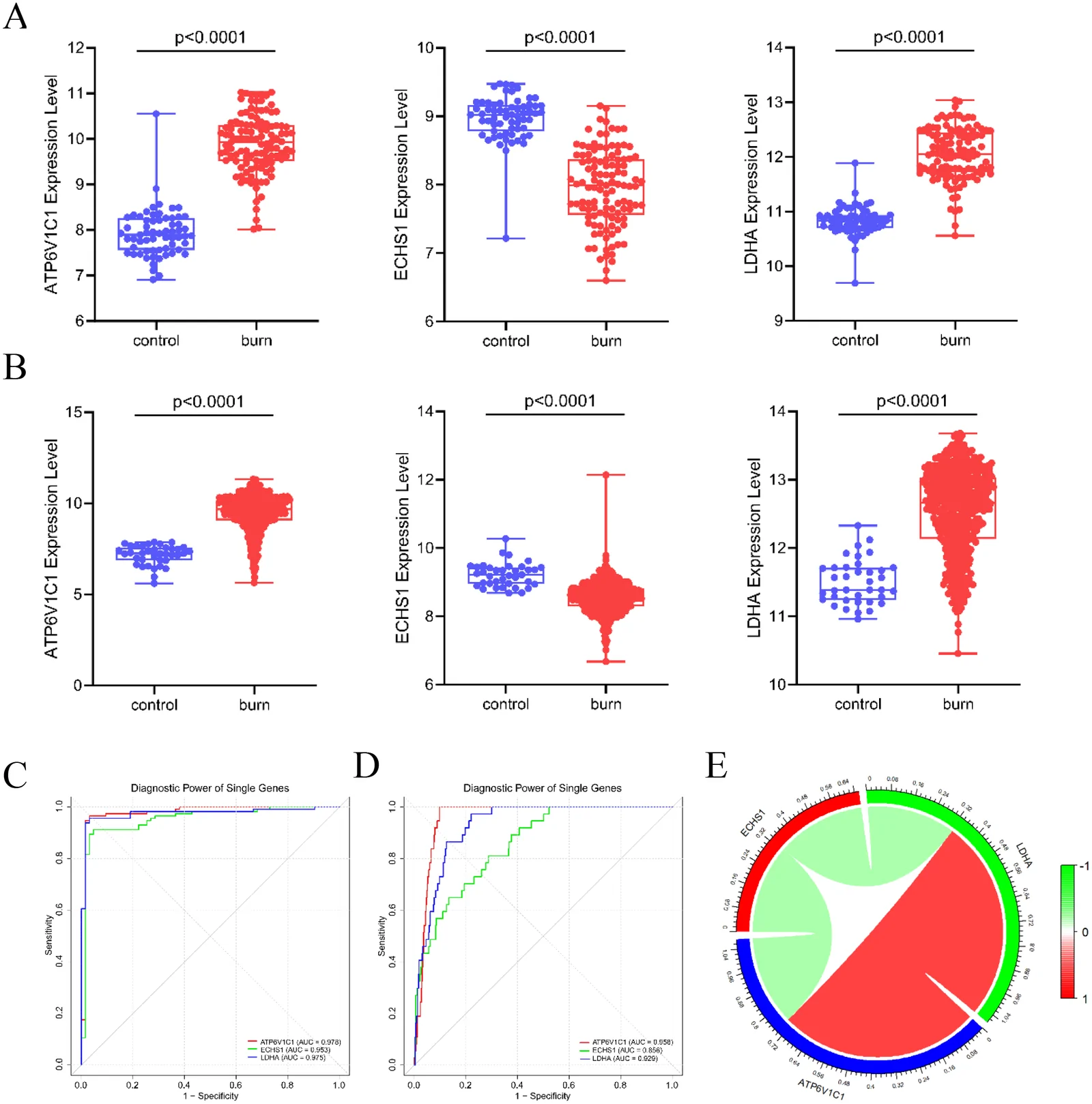
Validation and correlation analysis of core genes in burn patients. **(A)** Expression of ATP6V1C1, ECHS1, and LDHA in burn and normal samples from the training set (GSE37069). ATP6V1C1 and LDHA are significantly upregulated, while ECHS1 is downregulated in burn samples. **(B)** Expression of ATP6V1C1, ECHS1, and LDHA in burn and normal samples from the test set (GSE19743), showing similar trends as in the training set. **(C)** ROC curve analysis of ATP6V1C1, ECHS1, and LDHA in the training set (GSE37069). AUC values for ATP6V1C1, ECHS1, and LDHA are 0.978, 0.953, and 0.975, respectively. **(D)** ROC curve analysis of ATP6V1C1, ECHS1, and LDHA in the test set (GSE19743), with AUC values of 0.958, 0.856, and 0.929, respectively. **(E)** Chord diagram representing the correlations between ATP6V1C1, ECHS1, and LDHA, where green lines indicate negative correlations and red lines indicate positive correlations. ECHS1 is negatively correlated with both ATP6V1C1 and LDHA, while ATP6V1C1 and LDHA are positively correlated.

Next, we validated the expression differences of the three core genes between burn and normal samples in two independent datasets. In the training set (GSE37069), the expression levels of ATP6V1C1 and LDHA were significantly higher in burn samples compared to normal samples, while the expression of ECHS1 was significantly lower in burn samples (**Figure 3A**). Similarly, in the test set (GSE19743), we observed the same trend, with ATP6V1C1 and LDHA expression levels significantly higher in burn samples, and ECHS1 expression significantly lower (**Figure 3B**). To further assess the diagnostic performance of these three genes in burn patients, we calculated their area under the ROC curve (AUC). In the training set (GSE37069), the AUC for ATP6V1C1 was 0.978, ECHS1 was 0.953, and LDHA was 0.975 (**Figure 3C**), indicating strong diagnostic power. In the test set (GSE19743), the AUC for ATP6V1C1 was 0.958, ECHS1 was 0.856, and LDHA was 0.929 (**Figure 3D**), which also showed high diagnostic value, further confirming the model’s excellent generalizability. We then analyzed the correlations between these three core genes. A chord diagram was used to represent the correlations, with green indicating negative correlations and red indicating positive correlations. The results showed significant negative correlations between ECHS1 and both ATP6V1C1 and LDHA, while ATP6V1C1 and LDHA showed a significant positive correlation (**Figure 3E**). This suggests that these three genes may be closely interconnected and collaboratively regulate the progression of burn injury in patients.

### 3.3 Construction and validation of the nomogram

**Figure 4:**
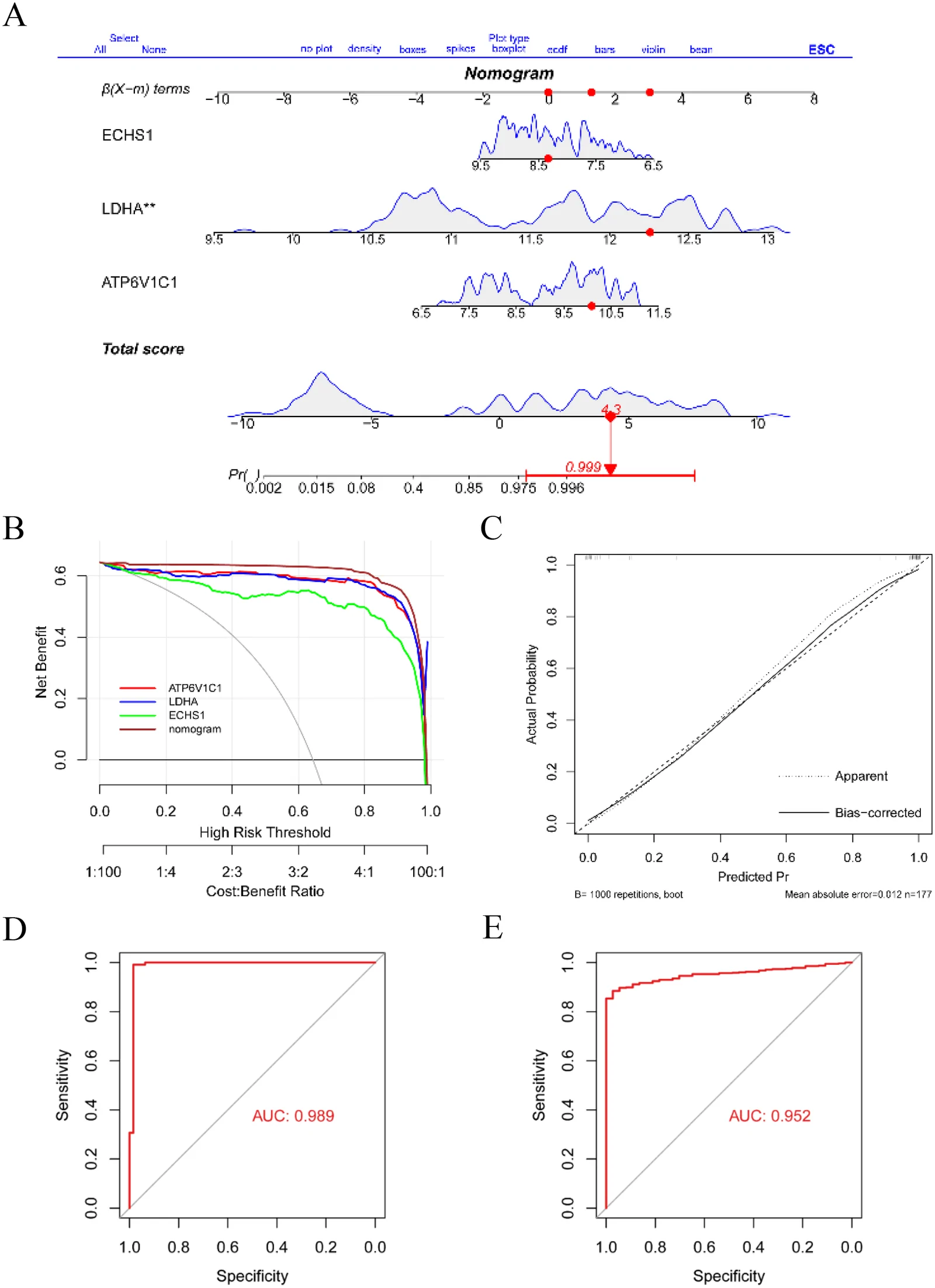
Nomogram construction and validation. **(A)** Nomogram for burn diagnosis based on the logistic regression model using ATP6V1C1, ECHS1, and LDHA gene expression. **(B)** Decision curve analysis **(DCA)** comparing the clinical net benefit of the burn diagnosis model versus single gene models. **(C)** Calibration curve showing the good consistency between predicted probabilities and actual probabilities. **(D)** ROC curve of the burn diagnosis model in the training set (GSE37069) with an AUC of 0.989. **(E)** ROC curve of the burn diagnosis model in the independent test set (GSE19743) with an AUC of 0.952.

To further explore the diagnostic value of the three core genes in burn patients, we constructed a burn diagnosis model based on a logistic regression framework, incorporating ATP6V1C1, LDHA, and ECHS1 genes. The regression results were visualized using a Nomogram (**Figure 4A**). To evaluate the clinical utility of this model, decision curve analysis (DCA) was performed, showing that the model provided higher clinical net benefit compared to individual genes, further demonstrating its potential for practical clinical application (**Figure 4B**). Additionally, calibration curves indicated a good consistency between the predicted and actual probabilities (**Figure 4C**). In the training set (GSE37069), the model exhibited excellent discriminatory power, with an area under the ROC curve (AUC) of 0.989 (**Figure 4D**). In the independent validation set (GSE19743), the model demonstrated stable predictive performance, with an AUC of 0.952 (**Figure 4E**), confirming its robustness and generalizability. Moreover, univariable logistic regression showed that each of the three core genes was strongly associated with burn injury (all P < 1×10^-9^); in the multivariable model, LDHA remained an independent predictor (OR = 30.92, 95% CI 3.12–305.97, P = 0.003), whereas the associations of ATP6V1C1 and ECHS1 were attenuated by their strong intercorrelation (Pearson |r| = 0.57–0.76), consistent with the coordinated regulation of these oxidative phosphorylation–related genes and supporting their combined use in a single diagnostic model (**Supplementary Table 2**).

### 3.4 Consensus clustering of burn patients

**Figure 5:**
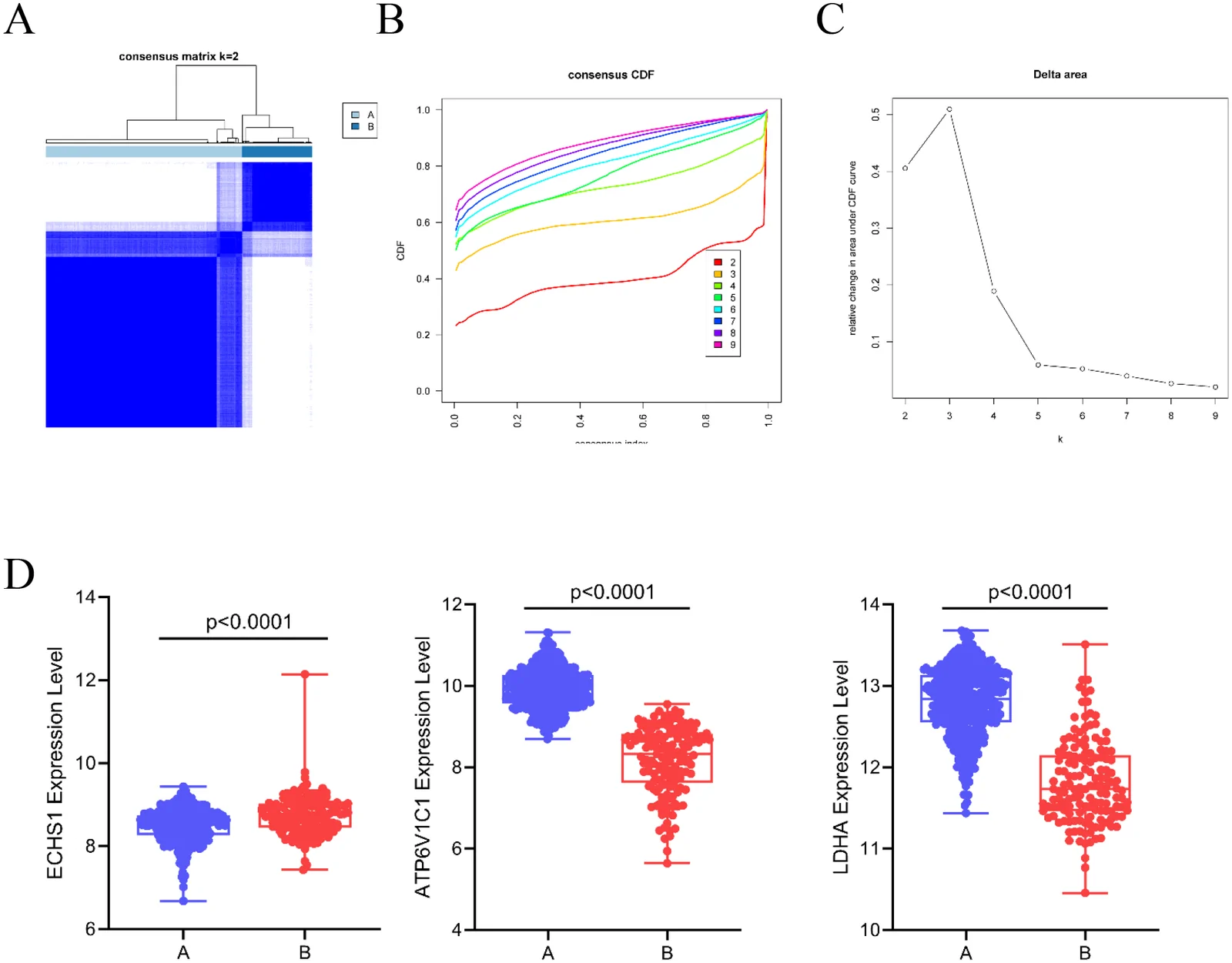
Consensus clustering and expression patterns of core genes in burn patients. **(A-C)** Consensus clustering of burn patients based on the expression of ATP6V1C1, ECHS1, and LDHA, with k=2 showing the best intra-group correlation and low inter-group correlation, indicating the presence of two distinct subgroups. **(D)** Expression patterns of ATP6V1C1, ECHS1, and LDHA in the two subgroups **(A, B)**, with significant differences in gene expression between the two groups. ECHS1 is lower in Group A, while ATP6V1C1 and LDHA are higher in Group A.

To explore the relationship between the three core genes and the classification of burn patients, we performed consensus clustering analysis based on the expression patterns of the three core genes. We incrementally increased the number of clusters (k) from 2 to 10, and the results showed that when k=2, the intra-group correlation was highest while the inter-group correlation was low, indicating that burn patients could effectively be divided into two distinct clusters based on the expression of these three genes (**Figures 5A–C**). Next, we analyzed the expression patterns of the three core genes in the two subgroups. We found that the expression of ECHS1 in Group A was significantly lower than in Group B, while ATP6V1C1 and LDHA expression were significantly higher in Group A compared to Group B (**Figure 5D**). This suggests that there are significant differences in the expression patterns of the three core genes between the two subgroups.

### 3.5 Subgroup functional clustering and immune infiltration analysis in burn patients

**Figure 6:**
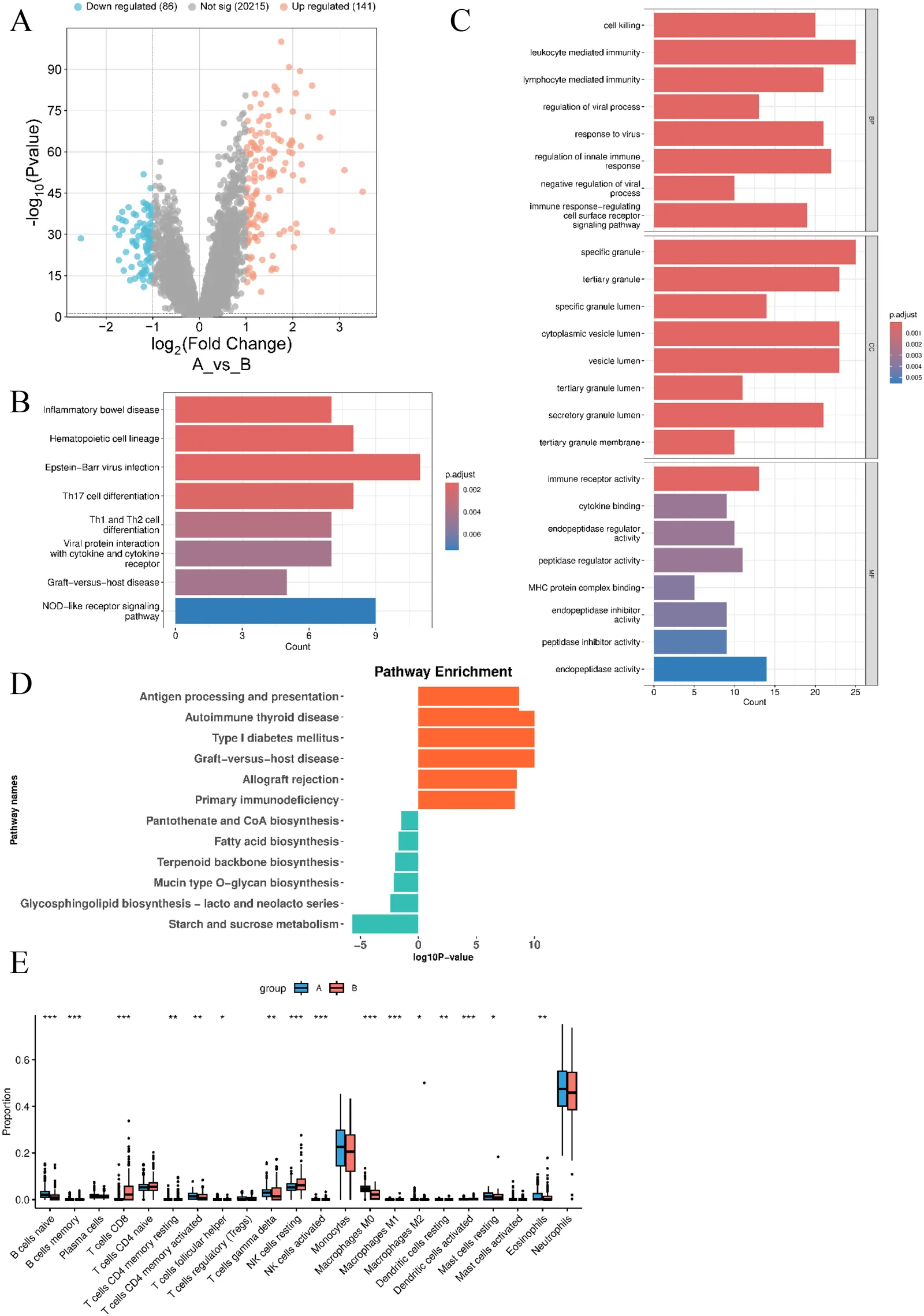
Functional and immune infiltration analysis of subgroups in burn patients. **(A)** Differential gene expression analysis between Group A and Group B in burn patients. A total of 227 differentially expressed genes were identified, including 86 downregulated and 141 upregulated genes. **(B)** KEGG pathway analysis of differentially expressed genes, highlighting enriched pathways such as “Inflammatory bowel disease”, “Hematopoietic cell lineage”, and “Th17 cell differentiation”. **(C)** GO analysis of differentially expressed genes, showing enrichment in immune-related functions like “leukocyte-mediated immunity”, “immune receptor activity”, and “cytokine binding”. **(D)** GSEA analysis revealing the most significantly activated KEGG pathways in Group A (positive enrichment score) and Group B (negative enrichment score). **(E)** Immune infiltration analysis using the CIBERSORT algorithm, showing significant differences in the levels of various immune cells between Group A and Group **(B)** Group A has higher levels of B cells, M0 macrophages, and CD4+ memory-activated T cells, while Group A has lower levels of CD8+ T cells, CD4+ memory resting T cells, and resting NK cells compared to Group **(B)** For clarity, only the immune cell subsets showing statistically significant differences between groups are described in the main text. Differences in immune cell infiltration were assessed using the Wilcoxon rank-sum test.

To investigate the gene expression and functional differences between Group A and Group B burn patients, we performed differential gene expression analysis using the limma package, with thresholds set at |log2FC| > 1 and P-value < 0.05. This analysis identified 227 differentially expressed genes, including 86 downregulated and 141 upregulated genes (**Figure 6A**). We then conducted KEGG and GO analyses on these 227 genes. KEGG analysis revealed that these genes were primarily enriched in pathways related to “Inflammatory bowel disease”, “Hematopoietic cell lineage”, “Epstein-Barr virus infection”, and “Th17 cell differentiation” (**Figure 6B**). GO analysis further indicated that these genes were mainly involved in immune-related functions such as “leukocyte-mediated immunity”, “immune receptor activity”, and “cytokine binding” (**Figure 6C**). These findings suggest that the differential genes between Group A and Group B are closely associated with immune regulation.

Next, we performed GSEA (Gene Set Enrichment Analysis) to further identify the most significantly different KEGG pathways between Group A and Group B. Genes were ranked by |log2FC| from smallest to largest, and GSEA revealed that “Antigen processing and presentation”, “Autoimmune thyroid disease”, and “Type I diabetes mellitus” pathways were significantly activated in Group A (positive enrichment score), whereas in Group B, pathways like “Starch and sucrose metabolism”, “Glycosphingolipid biosynthesis − lacto and neolacto series”, and “Mucin type O−glycan biosynthesis” were significantly activated (negative enrichment score) (**Figure 6D**). Given that multiple immune-related pathways were significantly activated in Group A and showed marked differences from Group B, we further quantified the infiltration levels of 22 immune cell subsets in the two groups using the CIBERSORT algorithm. The results showed that the immune infiltration landscape differed significantly between the two groups, with widespread alterations in immune cell proportions. We focus here on the most markedly altered subsets with biological relevance. Specifically, Group A had higher proportions of naive B cells, M0 macrophages, and activated CD4+ memory T cells, whereas lower proportions of CD8+T cells, resting CD4+ memory T cells, and resting NK cells were observed in Group A compared with Group B (**Figure 6E**). These findings suggest that the three oxidative phosphorylation-related genes can effectively distinguish different subgroups of burn patients and may provide support for personalized treatment strategies for burn injury.

### 3.6 Correlation analysis of core genes and immune cell infiltration

**Figure 7:**
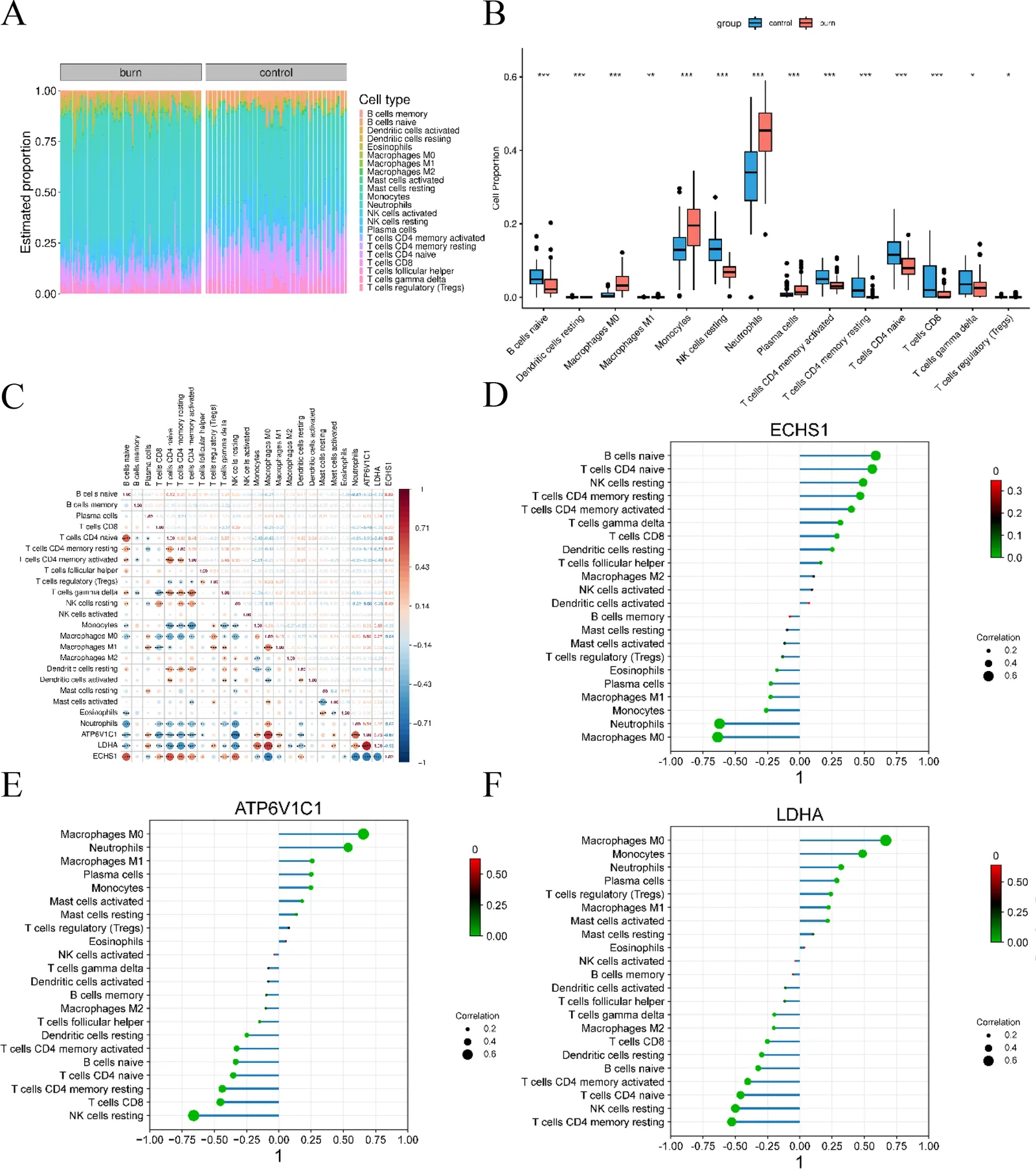
Correlation between core genes and immune cell infiltration in burn patients. **(A)** Heatmap showing the differences in immune cell infiltration between healthy and burn samples. **(B)** Immune cell composition analysis revealing significant changes in immune cell levels in burn patients, including increased monocytes, M0 macrophages, and neutrophils, and decreased naive B cells, naive CD4+ T cells, and activated CD4+ memory T cells. **(C)** Correlation analysis between the expression of core genes (ATP6V1C1, ECHS1, and LDHA) and immune cell levels. **(D)** Correlation between ECHS1 expression and immune cells, showing positive correlations with B cells, naive CD4+ T cells, and resting NK cells. **(E, F)** Correlation between ATP6V1C1 and LDHA expression and immune cells, showing positive correlations with monocytes, M0 macrophages, and neutrophils.

To further explore the relationship between the expression levels of the three core genes and immune infiltration in burn patients, we first analyzed the impact of burns on the immune microenvironment. The heatmap revealed significant differences in immune infiltration between healthy and burn samples (**Figure 7A**). A broad range of immune cell subsets showed significant proportion changes after burn injury. We describe only the most markedly altered subsets here: monocytes, M0 macrophages, and neutrophils were significantly increased after burns, while naive B cells, naive CD4+ T cells, and activated CD4+ memory T cells were significantly decreased (**Figure 7B**).

Next, we performed a correlation analysis between the expression levels of these immune cells and the three core genes (**Figure 7C**). Specifically, ECHS1 expression was positively correlated with B cells, naive CD4+ T cells, and resting NK cells (**Figure 7D**). This result is consistent with the previously observed downregulation of ECHS1 in burn patients, suggesting that ECHS1 may influence burn progression by modulating these immune cell levels. Similarly, ATP6V1C1 and LDHA expression levels were positively correlated with monocytes, M0 macrophages, and neutrophils (**Figure 7E, F**). These findings suggest that these three genes may regulate the immune microenvironment and, in turn, influence the progression of burn injury in patients.

### 3.7 Experimental validation of core gene expression in clinical burn samples

**Figure 8:**
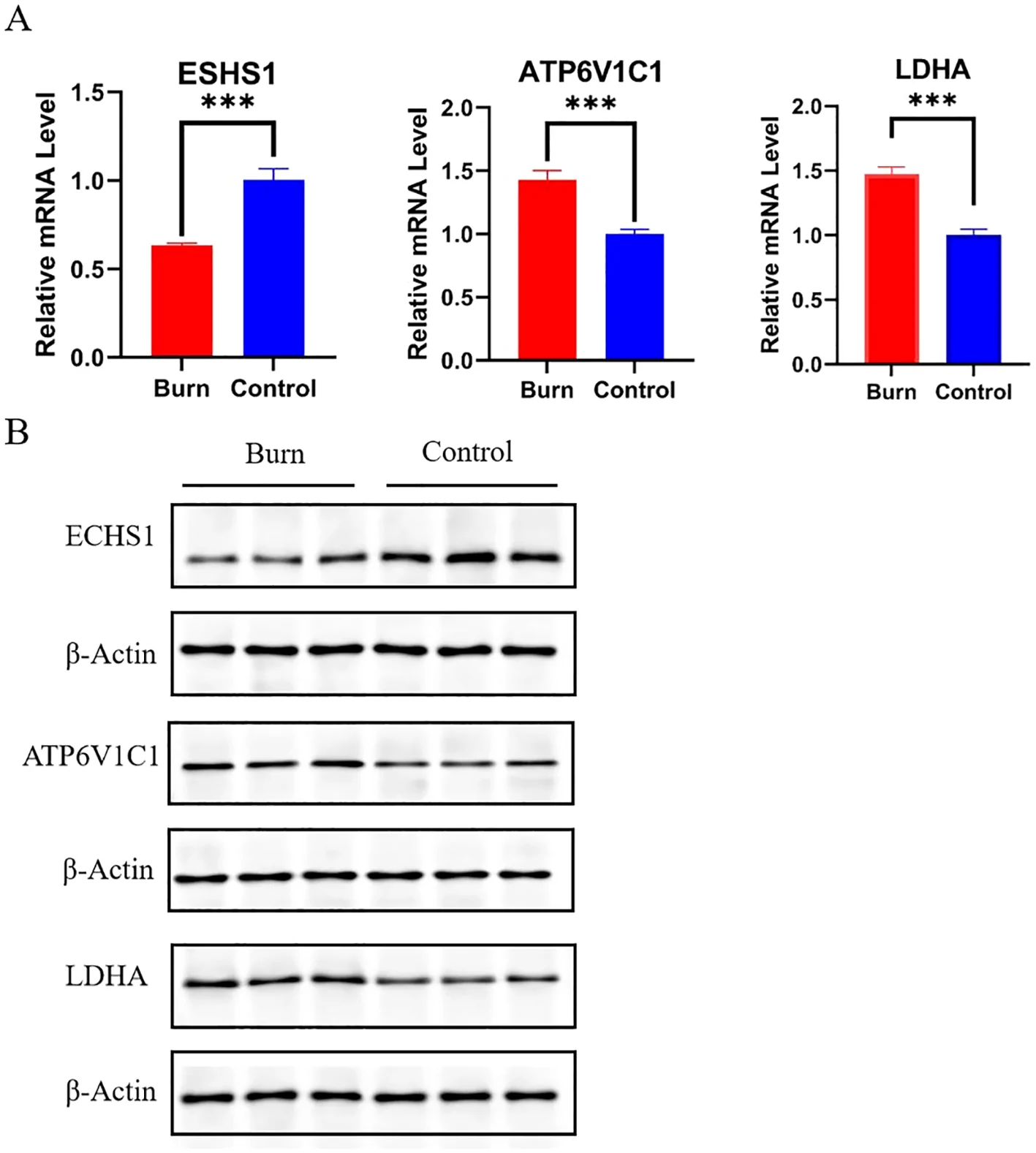
Experimental validation of core gene expression in clinical burn samples. **(A)** qPCR analysis of ATP6V1C1, ECHS1, and LDHA mRNA levels in burn and adjacent normal tissues, showing significant downregulation of ECHS1 and upregulation of ATP6V1C1 and LDHA in the burn group. **(B)** Western blot analysis of ATP6V1C1, ECHS1, and LDHA protein levels in burn and normal tissues with β-Actin as the loading control; ATP6V1C1 and LDHA are upregulated, whereas ECHS1 is downregulated in the burn group. Data are presented as mean ± SD (n = 20 pairs); statistical significance was determined by a paired two-tailed Student’s t test (***P < 0.001).

Next, we validated the expression of the three core genes in clinical burn tissue samples. First, we used qPCR to assess the gene expression differences between normal and burn tissues. As expected, we found that ECHS1 expression was significantly lower in the burn group compared to the normal group, while the expression of ATP6V1C1 and LDHA was significantly higher in the burn group (**Figure 8A**). The qPCR was performed on paired burn and adjacent normal tissues (n = 20 pairs; see **Supplementary Table 1** for the clinical characteristics of the cohort).

Furthermore, we performed WB analysis to examine the total protein levels of the three core genes. Consistent with the transcriptional results, the protein levels of ATP6V1C1 and LDHA were significantly higher in the burn group, whereas ECHS1 protein was significantly lower compared with the normal group (**Figure 8B**). Densitometric quantification normalized to β-Actin confirmed these differences (**Figures 8B**, right). These results demonstrate that, at both the mRNA and protein levels, ATP6V1C1 and LDHA are upregulated while ECHS1 is downregulated in burn tissues, which is fully consistent with the bioinformatic predictions.

### 3.8 Silencing of oxidative phosphorylation-related genes reshapes macrophage inflammatory cytokine production

**Figure 9:**
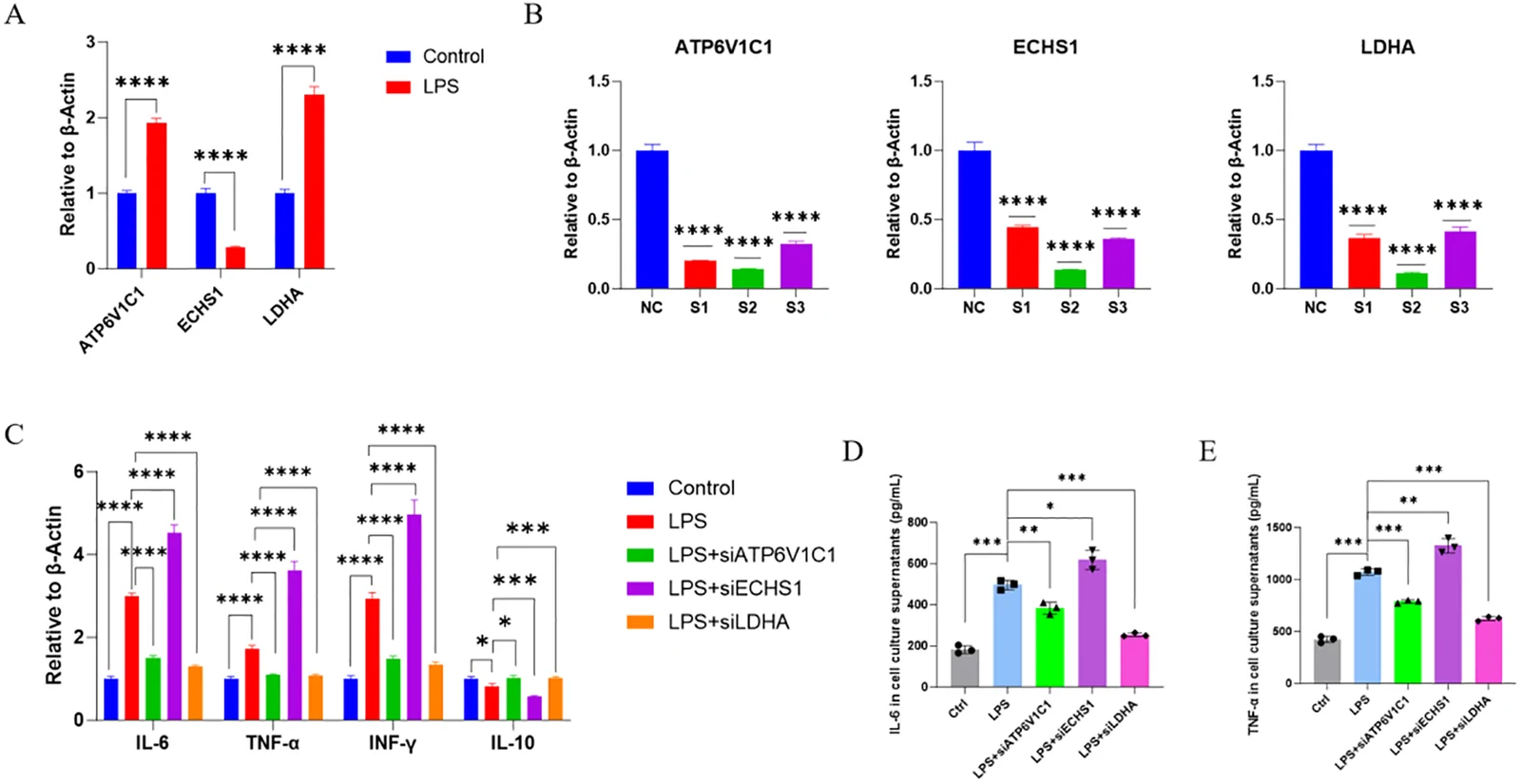
Silencing of oxidative phosphorylation–related genes modulates macrophage inflammatory cytokine production. **(A)** qPCR analysis of ATP6V1C1, ECHS1, and LDHA mRNA levels in PMA-differentiated THP-1 macrophages with or without LPS (100 ng/mL, 24 h) stimulation. **(B)** Knockdown efficiency of three siRNAs for each gene relative to the negative control (NC), assessed by qPCR. **(C)** qPCR analysis of IL-6, TNF-α, IFN-γ, and IL-10 mRNA levels in LPS-stimulated macrophages after transfection with the negative control or siRNA targeting ATP6V1C1, ECHS1, or LDHA. **(D, E)** ELISA quantification of secreted IL-6 **(D)** and TNF-α **(E)** in the culture supernatants of the corresponding groups. Data are presented as mean ± SD of three independent experiments; one-way ANOVA with Tukey’s *post hoc* test (*P < 0.05; **P < 0.01; ***P < 0.001; ****P < 0.0001).

To move from association to function and to clarify how the three oxidative phosphorylation–related genes participate in the post-burn immune response, we established an *in vitro* inflammatory model by differentiating THP-1 monocytes into macrophages with PMA and stimulating them with LPS (100 ng/mL, 24 h). LPS stimulation reproduced the expression pattern observed in the transcriptomic and clinical data: the mRNA levels of ATP6V1C1 and LDHA were markedly increased (approximately 1.9- and 2.3-fold, respectively), whereas ECHS1 was strongly decreased (to approximately 0.3-fold) relative to unstimulated controls (**Figure 9A**). Three siRNAs were designed for each gene, and the most efficient siRNA was selected for functional experiments after confirmation of knockdown efficiency by qPCR (**Figure 9B**).

We then examined the effect of silencing each gene on the production of key immune cytokines. At the transcriptional level, LPS strongly induced the pro-inflammatory cytokines IL-6, TNF-α, and IFN-γ; silencing ATP6V1C1 or LDHA significantly attenuated this induction, whereas silencing ECHS1 further increased their expression and reduced the anti-inflammatory cytokine IL-10 (**Figure 9C**). Consistent results were obtained at the protein level by ELISA: compared with LPS stimulation alone, knockdown of ATP6V1C1 or LDHA significantly reduced the secretion of IL-6 and TNF-α, whereas knockdown of ECHS1 further enhanced their secretion (**Figure 9D, E**). These findings indicate that ATP6V1C1 and LDHA promote, whereas ECHS1 restrains, the LPS-driven inflammatory activation of macrophages, providing direct functional support for the immune-regulatory roles inferred from the bioinformatic correlation analysis.

### 3.9 Silencing of oxidative phosphorylation-related genes alters mitochondrial oxidative metabolism

**Figure 10:**
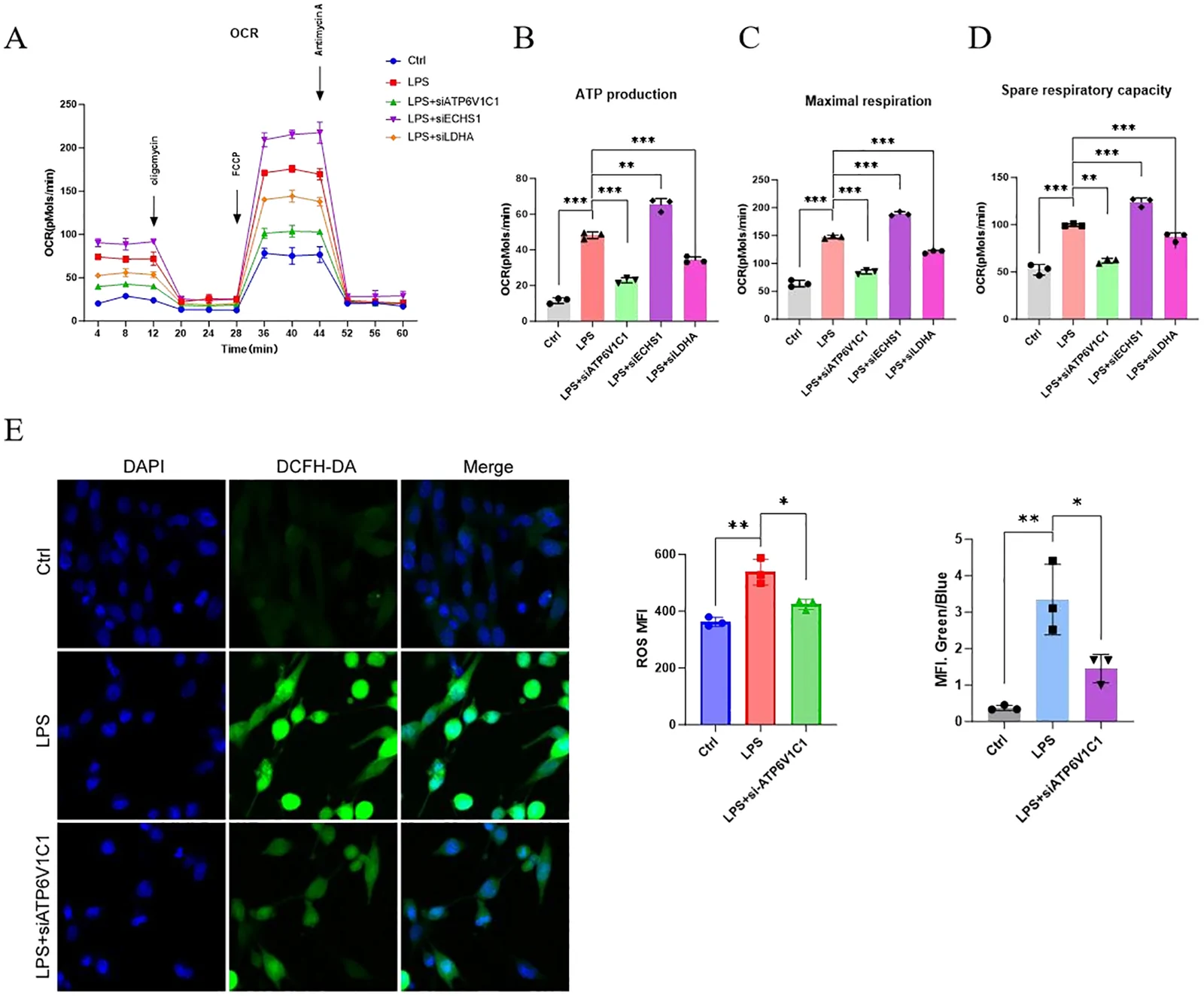
Silencing of oxidative phosphorylation–related genes alters mitochondrial oxidative metabolism in macrophages. **(A)** Real-time OCR profiles of LPS-stimulated THP-1 macrophages transfected with the negative control or siRNA targeting ATP6V1C1, ECHS1, or LDHA, measured with the Seahorse XF Cell Mito Stress Test. **(B–D)** Maximal respiration **(B)**, ATP production **(C)**, and spare respiratory capacity **(D)** derived from the OCR profiles. **(E)** Intracellular ROS levels measured by DCFH-DA staining and flow cytometry (mean fluorescence intensity). Data are presented as mean ± SD of three independent experiments; one-way ANOVA with Tukey’s *post hoc* test (*P < 0.05; **P < 0.01; ***P < 0.001).

Because oxidative phosphorylation is central to mitochondrial energy metabolism and to the metabolic reprogramming that accompanies macrophage activation, we next assessed the impact of gene silencing on mitochondrial function. Using the Seahorse XF Cell Mito Stress Test, we found that LPS stimulation markedly increased the oxygen consumption rate (OCR), including maximal respiration, ATP production, and spare respiratory capacity (**Figure 10A-D**). Silencing ATP6V1C1 strongly suppressed the LPS-induced increase in mitochondrial respiration, and silencing LDHA produced a partial reduction, whereas silencing ECHS1 further enhanced OCR (**Figure 10A-D**). In parallel, LPS stimulation substantially increased intracellular reactive oxygen species (ROS), and silencing ATP6V1C1 significantly reduced ROS accumulation (**Figure 10E**). Together, these data demonstrate that the three oxidative phosphorylation–related genes differentially regulate mitochondrial oxidative metabolism and ROS production in activated macrophages, linking their expression changes to the metabolic and immune reprogramming that occurs after burn injury.

## 4 Discussion

Oxidative phosphorylation (OxPhos) is a central process in cellular energy metabolism that not only directly regulates cellular energy supply but also plays a crucial role in modulating cellular stress responses, metabolic balance, and cell survival (12). It has been shown that OxPhos is vital for maintaining cellular function, especially following acute injuries such as burns, where metabolic dysregulation exacerbates mitochondrial dysfunction, leading to abnormal immune responses and delayed repair processes (13). Burn-induced extensive tissue damage necessitates substantial energy support, and mitochondrial dysfunction not only impairs the normal function of immune cells but also amplifies either an excessive immune reaction or immune tolerance during the repair process (14).

In this study, differential expression analysis identified key genes significantly associated with burns and further screened core genes related to the oxidative phosphorylation pathway. Functional enrichment analysis revealed that these genes are primarily involved in the regulation of mitochondrial function, particularly in pathways related to mitochondrial membranes, inner membranes, and mitochondrial protein complexes, suggesting that oxidative phosphorylation plays a pivotal role in tissue repair and immune responses following burns. Previous studies have also highlighted the close relationship between mitochondrial dysfunction and metabolic disorders after acute injury, especially during the early stages of burn injury, where disruption of oxidative phosphorylation may lead to insufficient energy supply, thereby affecting immune cell function and tissue repair (15). KEGG pathway enrichment analysis further indicated that key metabolic pathways, such as oxidative phosphorylation, pyruvate metabolism, and fatty acid degradation, are significantly associated with the metabolic disturbances and immune responses observed after burns. It has been suggested that oxidative phosphorylation not only serves as the core process for mitochondrial energy production but also interacts closely with energy-regulating pathways like fatty acid metabolism, thereby affecting both cellular metabolic balance and immune responses (16). These findings underscore the potential role of oxidative phosphorylation genes in metabolic and immune regulation after burns, providing new molecular targets for future therapeutic strategies.

It is worth noting that the immune response after burns is complex and dynamic, and changes in the immune microenvironment directly affect the patient’s recovery process. In addition to the key metabolic pathways identified through KEGG analysis, we further screened three core genes closely related to oxidative phosphorylation using Lasso, SVM-RFE, and Random Forest algorithms: ATP6V1C1, ECHS1, and LDHA. These genes not only play significant roles in energy metabolism but may also exert important effects on the immune response after burns by regulating immune cell function. ATP6V1C1 is a subunit of ATP synthase, involved in mitochondrial ATP synthesis, and is an important regulator of cellular energy metabolism (17). Previous studies have shown that ATP6V1C1 plays a critical role in cellular energy metabolism, especially under high-energy-demand conditions, where its expression changes may reflect the regulation of mitochondrial function (18). During burn injury, changes in ATP6V1C1 expression may help maintain energy balance, promoting wound healing and tissue repair. ECHS1 encodes an enzyme in the mitochondria involved in fatty acid metabolism (19). After burns, fatty acid metabolism plays a key role in cellular energy supply and immune response (20). Fatty acid oxidation is not only a critical pathway for energy production but also regulates immune cell activity. Previous studies have indicated that changes in ECHS1 may affect lipid metabolism and immune responses, thereby playing an important role in immune tolerance and repair after burns (21). Lactate dehydrogenase A (LDHA) is an enzyme involved in converting pyruvate to lactate in the glycolysis pathway (22). Studies have shown that LDHA responds to high metabolic demands and oxygen supply shortages under hypoxic conditions by regulating lactate metabolism (23). Therefore, LDHA may play a similar role in metabolic adaptation after burns. However, the specific regulatory mechanisms of LDHA during the burn process remain limited and require further exploration and validation.

Burns, as an acute injury, not only lead to local tissue damage but also trigger a systemic immune response. This response is characterized by an initial excessive inflammation, followed by an immunosuppressive state. The balance between these two phases is crucial for the recovery of burn patients (24, 25). Through immune infiltration analysis using the CIBERSORT algorithm, we found significant differences in the immune cell composition between Group A and Group B, especially in terms of the relative abundance of various cell types. The immune response in Group A was more active, with an increase in immune cells such as B cells, M0 macrophages, and CD4+ memory activated T cells, whereas the levels of CD8+ T cells and resting NK cells were lower. Group B, on the other hand, showed a lower level of immune activity. The more active immune response in Group A may be closely related to early immune clearance and tissue repair. During the early stages of burns, the activation and proliferation of immune cells are crucial for clearing dead cells, pathogens, and regulating inflammation. For instance, M0 macrophages can transform into different subtypes (e.g., M1 macrophages) to drive inflammatory responses and promote pathological clearance (26), while CD4+ memory activated T cells are involved in immune memory and specific responses to infections (27). Additionally, B cells play an important role in clearing damaged areas by producing antibodies during the inflammatory response. However, the reduced levels of CD8+ T cells and resting NK cells in Group A may suggest that these patients exhibit a certain degree of immune tolerance. CD8+ T cells are primarily responsible for killing tumor and virus-infected cells, while NK cells act as the “first line of defense” against infections and tumor cells. The enhanced immune tolerance in Group A may be a mechanism to prevent an overactive immune response, thus avoiding damage to normal tissues, especially after burns, where excessive immune reactions can lead to chronic inflammation and tissue necrosis (28). In contrast, patients in Group B exhibited weaker immune responses, potentially indicating immune evasion. This state signifies that the immune system’s suppressive function is too pronounced, leading to the failure to effectively eliminate damaged cells or repair injuries. Immune evasion is often characterized by impaired T cell function, reduced macrophage reactivity, or enhanced immune tolerance. This state allows pathogens and damaged cells to evade immune surveillance, delaying or suppressing tissue repair, and increasing the risk of secondary infections and organ failure (29).

Further analysis revealed a significant association between ECHS1 and immune cell subpopulations, highlighting its potential role in regulating immune responses. ECHS1 gene expression was positively correlated with the levels of naive B cells, CD4+ naive T cells, and resting NK cells, suggesting that ECHS1 may play an important role in enhancing the activity of these cells, thereby influencing the immune response after burn injury. These immune cells are crucial for immune repair in burn patients, and their activity is regulated by various cytokines and metabolic factors, which, in turn, affect wound healing and immune clearance (30). In contrast, the expression of ATP6V1C1 and LDHA was positively correlated with the levels of monocytes, M0 macrophages, and neutrophils, indicating that these genes may play a role in the early immune response. Macrophages, in particular, serve as “sentinels” and “cleaners” during the early stages of burns, responsible for clearing dead cells and pathogens and initiating subsequent repair processes (31). At the same time, the expression of LDHA is closely linked to metabolic pathways, and it may influence immune cell function by altering the metabolic state of cells. For instance, LDHA is involved in lactate production, and lactate can modulate immune responses, particularly in hypoxic environments. This may aid immune cell survival and function after burns (32). The dynamic changes in the immune microenvironment play a crucial role in recovery post-burn. Early immune clearance helps reduce the risk of infection and accelerates wound healing, while the maintenance of immune tolerance prevents overactive immune responses, thereby protecting healthy tissues from secondary damage. However, the emergence of immune evasion can lead to immune dysregulation, which impairs the repair process following burns. By regulating key genes such as ECHS1, ATP6V1C1, and LDHA, it may be possible to modulate the immune microenvironment balance and promote recovery in burn patients (33).

Importantly, our *in vitro* functional experiments moved beyond correlation and directly demonstrated the immunometabolic roles of these three genes. In LPS-activated macrophages, silencing ATP6V1C1 or LDHA suppressed the production of pro-inflammatory cytokines (IL-6, TNF-α, and IFN-γ) and dampened mitochondrial respiration and ROS generation, whereas silencing ECHS1 amplified the inflammatory response. These observations are consistent with the immune infiltration data, in which ATP6V1C1 and LDHA were positively correlated with the pro-inflammatory monocytes, M0 macrophages, and neutrophils that predominate in the more inflammatory Group A, whereas ECHS1 was associated with the B cells, naive CD4+ T cells, and resting NK cells that characterize the more tolerant Group B. Mechanistically, oxidative phosphorylation and glycolysis-linked lactate metabolism are increasingly recognized as key drivers of macrophage polarization and cytokine output (34); thus, the burn-induced upregulation of ATP6V1C1 and LDHA together with the downregulation of ECHS1 may reprogram macrophage metabolism toward a pro-inflammatory state, while the loss of ECHS1 removes a metabolic brake on inflammation. Comparable coupling between mitochondrial metabolism and immune activation has been reported in other acute inflammatory and traumatic conditions, suggesting that this represents a conserved cellular stress-response mechanism.

Several limitations of this study should be acknowledged. First, the GSE37069 and GSE19743 datasets comprise longitudinal blood samples collected at multiple time points from a limited number of severely burned patients rather than from independent individuals; although one dataset was used for training and the other for external validation, treating repeated measurements as independent samples may inflate statistical significance and model performance, and larger patient-level cohorts are needed for confirmation. Second, the current diagnostic model distinguishes severe burn patients from healthy controls but does not yet address clinically important questions such as burn-severity stratification, prediction of infection or sepsis, delayed wound healing, or the risk of multiple organ dysfunction syndrome; these will be the focus of future work. Third, the functional experiments were conducted in a THP-1 macrophage model, and validation in primary immune cells and in *in vivo* burn models will be required. Future studies will therefore focus on cell-type-specific and *in vivo* interventions and on the mechanistic dissection of the identified pathways.

In summary, the alterations in the immune microenvironment in burn patients are closely linked to immune cell activity and function, a process regulated by oxidative phosphorylation genes. This highlights the complex interactions between immune cells and core genes. These findings provide new perspectives and potential targets for immunotherapy in burns. Collectively, this study, through a detailed analysis of oxidative phosphorylation genes and their role in the post-burn immune microenvironment, elucidates their potential involvement in metabolic regulation and immune responses after burns. Future studies could focus on these key genes, exploring their regulatory mechanisms and providing theoretical support for individualized burn treatments. By precisely intervening in these genes and their signaling pathways, new therapeutic strategies may emerge to improve immune responses and tissue repair, thereby enhancing the diagnosis and prognosis of burn patients.

## Data Availability

The datasets presented in this study can be found in online repositories. The names of the repository/repositories and accession number(s) can be found in the article/supplementary material.
