# Supplementary material for: Identification and validation of core oxidative phosphorylation-related genes as biomarkers for immune response and diagnosis in burn injury
Source: Front Immunol. 2026 Aug 12;17:1818811. doi: 10.3389/fimmu.2026.1818811 (PMC13506282; doi:10.3389/fimmu.2026.1818811)
Supplement: Supplementary file 1 [file Presentation1.pdf]

Supplementary Information

dentification and validation of core oxidative phosphorylation-related genes as biomarkers for immune response and diagnosis in burn injury

Jiaqi Xu*[1]; Yujie Cui[2]; Kangdi Li[1];Jia Jia*[1]; Xinfeng Huang*[1];
(**Jiaqi Xu***, Yujie Cui and **Kangdi Li** have equal contributions)
[1]Burn and plastic surgery, the fifth affiliated hospital sun yat-sen university,ZhuHai,China;
[2] Rheumatology, the fifth affiliated hospital sun yat-sen university, ZhuHai,China;
*** Correspondence:**
Jiaqi Xu*
xujq33@mail.sysu.edu.cn
Jia Jia*
634354786@qq.com
Xinfeng Huang*
324008877@qq. Com

Supplementary Table S1.

Part 1: Demographic and clinical characteristics of enrolled subjects (n = 20 patients)

ID	Group	Age	Sex	Burn depth	%TBSA	Time from injury to sampling	Anatomical site	Infection status
B01	Burn	30	Male	II-III degree	42	< 24 h	Trunk + extremities	None
B02	Burn	58	Female	II degree	18	< 24 h	Trunk	None
B03	Burn	26	Female	II degree	11	< 24 h	Lower extremity	None
B04	Burn	32	Male	II degree	13	< 24 h	Back	None
B05	Burn	49	Male	II-III degree	20	< 24 h	Lower extremity	None
B06	Burn	54	Female	II degree	15	< 24 h	Shoulder + upper extremity	None
B07	Burn	59	Male	II degree	9	< 48 h	Lower extremity	None
B08	Burn	52	Male	II-III degree	25	< 48 h	Trunk + extremities	Local infection
B09	Burn	23	Female	II degree	8	< 24 h	Lower extremity	None
B10	Burn	51	Female	II degree	12	< 48 h	Lower extremity	None

ID	Group	Age	Sex	Burn depth	%TBSA	Time from injury to sampling	Anatomical site	Infection status
B11	Burn	55	Female	II degree	16	< 24 h	Chest and abdomen	None
B12	Burn	35	Male	II-III degree	30	< 24 h	Upper extremity + trunk	None
B13	Burn	38	Male	II degree	17	< 48 h	Back	None
B14	Burn	26	Male	II degree	10	< 24 h	Shoulder + upper extremity	None
B15	Burn	39	Male	II-III degree	22	< 24 h	Upper extremity	None
B16	Burn	71	Male	II-III degree	28	< 48 h	Multiple sites	None
B17	Burn	28	Male	II degree	19	< 24 h	Multiple sites	None
B18	Burn	37	Female	II degree	14	< 24 h	Multiple sites	None
B19	Burn	27	Male	II degree	10	< 24 h	Multiple sites	None
B20	Burn	26	Male	II-III degree	25	< 48 h	Multiple sites	None
N01	Control	44	Male	NA	NA	NA	Chest wall	None
N02	Control	24	Female	NA	NA	NA	Back	None

ID	Group	Age	Sex	Burn depth	%TBSA	Time from injury to sampling	Anatomical site	Infection status
N03	Control	41	Male	NA	NA	NA	Neck	None
N04	Control	58	Male	NA	NA	NA	Right upper extremity + lower back	None
N05	Control	59	Male	NA	NA	NA	Shoulder	None
N06	Control	43	Female	NA	NA	NA	Whole body	None
N07	Control	30	Male	NA	NA	NA	Thigh	None
N08	Control	35	Female	NA	NA	NA	Upper extremity	None
N09	Control	37	Female	NA	NA	NA	Abdominal wall	None
N10	Control	60	Female	NA	NA	NA	Back	None
N11	Control	55	Male	NA	NA	NA	Upper extremity	None
N12	Control	42	Male	NA	NA	NA	Back	None
N13	Control	40	Male	NA	NA	NA	Scalp	None
N14	Control	50	Male	NA	NA	NA	Back	None
N15	Control	60	Male	NA	NA	NA	Upper extremity	None
N16	Control	32	Male	NA	NA	NA	Multiple sites	None
N17	Control	53	Female	NA	NA	NA	Shoulder	None

ID	Group	Age	Sex	Burn depth	%TBSA	Time from injury to sampling	Anatomical site	Infection status
N18	Control	37	Female	NA	NA	NA	Subcutaneous tissue	None
N19	Control	31	Male	NA	NA	NA	Back	None
N20	Control	36	Female	NA	NA	NA	Back	None

Note: NA = Not applicable. All control subjects underwent elective surgery for benign skin lesions, with no history of infection, malignancy, or immune disorders.

Part 2: Demographic and clinical characteristics of the Public GEO transcriptomic cohorts.

Dataset composition.

Dataset	Platform	Burn samples	Control samples	Role	Sample structure
**GSE37069**	Affymetrix HG-U133 Plus 2.0	553	37	Training set	Longitudinal blood samples collected at multiple time points from severely burned patients (repeated measures)
**GSE19743**	Affymetrix HG-U133 Plus 2.0	114	63	External validation	114 arrays from 57 patients at two time points (Early/Mid) plus 63 healthy controls

Detailed characteristics of the GSE19743 cohort (individual-level metadata from the GEO series matrix).

Characteristic	Burn (n = 114 arrays)	Control (n = 63)
**No. of individuals**	57 patients (2 time points each)	63
**Age, years (mean ± SD)**	23.9 ± 20.1	20.8 ± 15.0
**Age range, years**	0–69	7–63
**Age group (Adult / Pediatric)**	62 / 52	28 / 35
**Sex (Male / Female)**	90 / 24	30 / 33
**Total body surface area burned, % (mean ± SD)**	66.4 ± 15.9	–
**sTBSA range, %**	40–93	–
**Time after injury, hours (median, range)**	246.4 (4.8–760.6)	–
**Sampling time group (Early / Mid)**	57 / 57	–
**Inhalation injury (Yes / No)**	74 / 40	–

**Survival (Yes / No)**	84 / 30	–

SD, standard deviation; TBSA, total body surface area. Data for GSE19743 were extracted directly from the GEO series matrix (GSE19743_series_matrix). Because the burn arrays represent two time points per patient, the samples are not fully independent; this is addressed in the Discussion (Limitations). Detailed individual-level demographics were not available in the deposited metadata for GSE37069.

Supplementary Table S2. Univariable and multivariable logistic regression of the three core oxidative phosphorylation-related genes for the diagnosis of burn injury.

Gene	Univariable OR (95% CI)	Univariable P	Multivariable OR (95% CI)	Multivariable P
**ATP6V1C1**	96.59 (22.48–415.05)	8.0×10^{-10}	4.95 (0.54–45.73)	0.158
**ECHS1**	0.02 (0.01–0.07)	4.9×10^{-10}	0.30 (0.06–1.56)	0.151
**LDHA**	130.44 (27.38–621.47)	9.6×10^{-10}	30.92 (3.12–305.97)	0.003

OR, odds ratio; CI, confidence interval. Logistic regression was performed on the GSE19743 cohort (114 burn vs 63 control); each gene was standardized before modelling, so ORs are expressed per 1-standard-deviation increase in log2 expression. In univariable analysis, all three genes were strongly associated with burn injury. In the multivariable model, LDHA remained an independent predictor, whereas the associations of ATP6V1C1 and ECHS1 were attenuated owing to the strong intercorrelation among the three co-regulated genes (Pearson |r| = 0.57–0.76); this supports combining the three genes into a single diagnostic model (nomogram) rather than interpreting them as fully independent markers.

Supplementary Table S3. Sequences of small interfering RNAs (siRNAs) used in this study

siRNA	Forward Sequences (5'-3')	Reverse Sequences (5'-3')
hLDHA-1046-si1	GAAGAAGAGUGCAGAUACA/dT//dT/	UGUAUCUGCACUCUUCUUC/dT//dT/
hLDHA-497-si2	GCUUAUUGUUUCAAAUCCA/dT//dT/	UGGAUUUGAAACAAUAAGC/dT//dT/
hLDHA-158-si3	GAAUAAGAUUACAGUUGUU/dT//dT/	AACAACUGUAAUCUUAUUC/dT//dT/

hECHS1-785-si1	GAAGUAAGUUGGAGAAGAA/dT//dT/	UUCUUCUCCAACUUACUUC/dT//dT/
hECHS1-1246-si2	GAUGCUGAUUAAAGUGAUA/dT//dT/	UAUCACUUUAAUCAGCAUC/dT//dT/
hECHS1-212-si3	GCCUGAUUGACGAGCUCAA/dT//dT/	UUGAGCUCGUCAAUCAGGC/dT//dT/
hATP6V1C1-252-si1	CGGCAACUUCAAAGAACAAUA/dT//dT/	UAUUGUUCUUUGAAGUUGCCG/dT//dT/
hATP6V1C1-398-si2	GCUCAAUACAUGGCUGAUGUA/dT//dT/	UACAUCAGCCAUGUAUUGAGC/dT//dT/
hATP6V1C1-422-si3	GGAAGAUAGCAAAGACAAA/dT//dT/	UUUGUCUUUGCUAUCUUCC/dT//dT/
